# A Classification Approach to Determine Cognitive Impairment Stage from Metabolomic Data

**DOI:** 10.1093/geroni/igaf122.3516

**Published:** 2025-12-31

**Authors:** Araya Cserepy, Joshua Chuah

**Affiliations:** Union College, Schenectady, New York, United States; Union College, Schenectady, New York, United States

## Abstract

Alzheimer’s Disease (AD) is a neurodegenerative disorder often marked by amyloid beta and tau accumulation, but metabolic changes can precede clinical onset by several years. Metabolites in particular have been implicated as early indicators of brain dysfunction. As such, this study applied machine learning to identify serum-derived metabolites distinguishing early (EMCI) from late mild cognitive impairment (LMCI). Using publicly available metabolite data from the Alzheimer’s Disease Neuroimaging Initiative (ADNI), we analyzed baseline profiles from 643 participants (222 EMCI, 421 LMCI) across 104 features. A two-stage feature selection framework combined univariate ANOVA F-testing (top 30 features) with multivariate recursive feature elimination (final 15 features) to find the most important metabolites for diagnosis. This feature selection framework was repeated across different training/testing set splits to ensure the reliability of the features selected. Random Forest classification with balanced weighting addressed dataset imbalance. Across all repetitions, a core panel of five metabolites emerged consistently across validation runs: glyceric acid, hyocholic acid (HCA), hyodeoxycholic acid (HDCA), tauro-muricholic acid (TAMCA), and succinic acid. These metabolites achieved 76% ± 3% accuracy on unseen validation data and 78% ± 6% on development data. Notably, the panel included bile acid metabolites (HCA, HDCA, TAMCA) and energy-related metabolites (glyceric acid, succinic acid), pointing to disruptions in the gut–liver axis and cellular metabolism during disease progression. Overall, this work highlights metabolic alterations that accompany the transition from EMCI to LMCI and demonstrates the potential of machine learning–based metabolomics for early detection and diagnosis in AD.

